# Dual Coding with STDP in a Spiking Recurrent Neural Network Model of the Hippocampus

**DOI:** 10.1371/journal.pcbi.1000839

**Published:** 2010-07-01

**Authors:** Daniel Bush, Andrew Philippides, Phil Husbands, Michael O'Shea

**Affiliations:** Centre for Computational Neuroscience and Robotics, University of Sussex, Falmer, Brighton, United Kingdom; RIKEN Brain Science Institute, Japan

## Abstract

The firing rate of single neurons in the mammalian hippocampus has been demonstrated to encode for a range of spatial and non-spatial stimuli. It has also been demonstrated that phase of firing, with respect to the theta oscillation that dominates the hippocampal EEG during stereotype learning behaviour, correlates with an animal's spatial location. These findings have led to the hypothesis that the hippocampus operates using a dual (rate and temporal) coding system. To investigate the phenomenon of dual coding in the hippocampus, we examine a spiking recurrent network model with theta coded neural dynamics and an STDP rule that mediates rate-coded Hebbian learning when pre- and post-synaptic firing is stochastic. We demonstrate that this plasticity rule can generate both symmetric and asymmetric connections between neurons that fire at concurrent or successive theta phase, respectively, and subsequently produce both pattern completion and sequence prediction from partial cues. This unifies previously disparate auto- and hetero-associative network models of hippocampal function and provides them with a firmer basis in modern neurobiology. Furthermore, the encoding and reactivation of activity in mutually exciting Hebbian cell assemblies demonstrated here is believed to represent a fundamental mechanism of cognitive processing in the brain.

## Author Summary

Changes in the strength of synaptic connections between neurons are believed to mediate processes of learning and memory in the brain. A computational theory of this synaptic plasticity was first provided by Donald Hebb within the context of a more general neural coding mechanism, whereby phase sequences of activity directed by ongoing external and internal dynamics propagate in mutually exciting ensembles of neurons. Empirical evidence for this cell assembly model has been obtained in the hippocampus, where neuronal ensembles encoding for spatial location repeatedly fire in sequence at different phases of the ongoing theta oscillation. To investigate the encoding and reactivation of these dual coded activity patterns, we examine a biologically inspired spiking neural network model of the hippocampus with a novel synaptic plasticity rule. We demonstrate that this allows the rapid development of both symmetric and asymmetric connections between neurons that fire at concurrent or consecutive theta phase respectively. Recall activity, corresponding to both pattern completion and sequence prediction, can subsequently be produced by partial external cues. This allows the reconciliation of two previously disparate classes of hippocampal model and provides a framework for further examination of cell assembly dynamics in spiking neural networks.

## Introduction

The hippocampus and surrounding medial temporal lobe are implicated in declarative memory function in humans and other mammals [1]. Electrophysiology studies in a range of species have demonstrated that the activity of single pyramidal cells within this region can encode for the presence of both spatial and non-spatial stimuli [2]. The majority of empirical investigation has focussed on place cells – neurons whose firing rate is directly correlated with an animal's spatial location within the corresponding place field [3]. Subsequent research has identified similar single cell responses to a variety of non-spatial cues including odour [4], complex visual images [5], [6], [7], running speed [8] and the concept of a bed or nest [9]. It has also been demonstrated that the exact timing of place cell discharge, relative to the theta oscillation which dominates the hippocampal EEG during learning, correlates with distance travelled through a place field [2], [7], [10]–[12]. This phase precession mechanism creates a compressed ‘theta coded’ firing pattern in place cells which corresponds to the sequence of place fields being traversed [13]. These findings have led to the hypothesis that the hippocampus operates using a dual rate and temporal coding system [14], [15]. Here we present a spiking neural network model which utilises a dual coding system in order to encode and recall both symmetric (auto-associative) and asymmetric (hetero-associative) connections between neurons that exhibit repeated synchronous and asynchronous firing patterns respectively.

The postulated mnemonic function of the hippocampus has been extensively modelled using recurrent neural networks, and this approach is supported by empirical data [16]–[19]. The biological correlate of these models is widely believed to be the CA3 region, which exhibits dense recurrent connectivity and wherein synaptic plasticity can be easily and reliably induced. Pharmacological and genetic knockout studies have demonstrated that NMDAr-dependent synaptic plasticity in CA3 is critical for the rapid encoding of novel information, and synaptic output from CA3 critical for its retrieval [20], [21]. Recurrent neural network models of hippocampal mnemonic function have generally utilised rate-coded Hebbian learning rules to generate reciprocal associations between neurons with concurrently elevated firing rates [22], [23]. Hypothetically, this corresponds to the presence of either multiple stimuli or multiple overlapping place fields encountered at a single location [24]–[27]. The hippocampus is also implicated in sequence learning, and temporally asymmetric plasticity rules have subsequently been employed in recurrent network models to generate hetero-associative connections between neurons that fire with repeated temporal correlation [28]–[38]. Hypothetically, this corresponds to a sequence of place fields being traversed or stimuli being encountered on a behavioural timescale [13]. Importantly, previous computational models of hetero-associative learning have typically encoded each successive stage of a learned sequence with the activity of a single neuron, while empirical studies estimate that place fields are typically encoded by an ensemble of several hundred place cells [2], [39]–[45]. No computational model has thus far integrated auto- and hetero- associative learning in order to simultaneously generate both bi-directional and asymmetric connections between neurons that are active at the same and successive theta phases respectively using a single temporally asymmetric synaptic plasticity rule.

Empirical data indicates that changes in the strength of synapses within the hippocampus can depend upon temporal correlations in pre- and post- synaptic firing according to a spike-timing dependent plasticity (STDP) rule [46]–[49]. It is not yet clear if rate-coded auto-associative network models of hippocampal mnemonic function are compatible with STDP or theta coded neural dynamics. Here, we examine the synaptic dynamics generated by several different STDP rules in a spiking recurrent neural network model of CA3 during the encoding of temporal, rate and dual coded activity patterns created by a phenomenological model of phase precession. We demonstrate that – under certain conditions - the STDP rule can generate both bi-directional connections between neurons which burst at concurrent theta phase and asymmetric connections between neurons which fire at consecutive theta phase. Subsequent superthreshold stimulation of a small number of simulated neurons generates putative recall activity, driven by recurrent excitation, that corresponds to pattern completion and/or sequence prediction in auto- and/or hetero- associative connections respectively. Interestingly, these neural dynamics are reminiscent of sharp wave ripple activity observed *in vivo* [50]–[54]. These findings demonstrate that STDP and theta coded neural dynamics are compatible with rate-coded auto-associative network models of hippocampal function. Furthermore, the encoding and reactivation of dual coded Hebbian phase sequences of activity in mutually exciting neuronal ensembles demonstrated here has been proposed as a general neural coding mechanism for cognitive processing [50], [55]–[60].

## Methods

### The Network Model

The neural network consists of *100* simulated excitatory neurons which, in the majority of simulations, are fully recurrently connected by single synapses except for self connections. Although the level of recurrent connectivity present in the CA3 region is estimated as *5–15%* (and is non-random), full recurrent connectivity has most often been employed in previous computational models of auto- associative learning [16]–[19], [39]. However, all simulations described here were also performed using networks with more realistic levels of recurrent connectivity (*15* separate pre-synaptic connections per simulated neuron, chosen from a random uniform distribution that excludes self-connections) and no significant differences were observed (data not shown).

### The Neuron Model

Simulated pyramidal cells operate according to the Izhikevich spiking model [61], which can replicate the firing patterns of all known types of cortical neurons with minimal computational complexity. The membrane potential (*v*) and a membrane recovery variable (*u*) are dynamically calculated based on the values of four dimensionless constants (*a*, *b*, *c* and *d*) and a dimensionless current input (*I*) according to Equations 1.1–1.3.
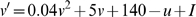
(1.1)

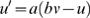
(1.2)

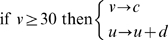
(1.3)The parameter values used to replicate firing of a standard excitatory neuron are [*a = 0.02, b = 0.2, c = −65, d = 6*]. Under these conditions, simulated neurons fire single spikes at low levels of stimulation, but produce complex bursts that are representative of hippocampal pyramidal cells (i.e. several action potentials at a spontaneous rate of ∼*150Hz*) at higher levels of stimulation [2], [62]. Further details of the dynamics produced by single simulated neurons in response to various forms of applied current can be found in Izhikevich (2004).

Each simulated neuron has an axonal delay (*D_i_*) randomly assigned from a uniform distribution in the range [*1ms : Dms*] with *D = 5* in the majority of simulations (this being realistic of the CA3 region [63]). At the beginning of each millisecond time step, before the parameters *v* and *u* are updated, any membrane potential values that exceed threshold are reset according to Equation 1.3. The corresponding neuron(s) are considered to have fired in that time step (t*), and the corresponding spikes arrive at their post-synaptic targets at time t*+D_i_.

### External Input during Theta Coded Learning

The hippocampal EEG is dominated by both theta and gamma oscillations during stereotype learning behaviour [39], [43], [64], [65]. Here, we include only a minimal model of theta frequency inhibition. A variable *θ*, which oscillates sinusoidally in the range [*0 : 1*] at a rate of *8Hz* throughout all learning simulations, is used to dynamically represent the theoretical local field potential (LFP). Inhibitory input to every simulated neuron at each millisecond time step is randomly sampled from a Gaussian distribution with mean *I_inh_ = −15θ* and standard deviation *σ_inh_ = 2*. Neural noise at a rate of ∼*0.1Hz* (this being realistic of the CA3 region) is generated in the network by the constant application of excitatory current, randomly sampled from a uniform distribution in the range [*0 : I_noise_*] where *I_noise_ = 0.8* in all simulations [66]. The interplay between afferent inhibitory and excitatory currents means that the majority of firing due to neural noise tends to occur around the peak of the LFP, as defined by the value of *θ*.

Place cells are most often studied in the dorsal CA1 region of the hippocampus, although some data is available from CA3 and, importantly, significant differences can be observed [14], [67]–[69]. Approximately *30%* of CA3 pyramidal cells are active in a typical environment, each of which can encode for several (occasionally overlapping) place fields of ∼*30*cm in size (although this varies along the septotemporal axis) [10], [12], [70]. The phase precession of place cell firing can cover a full theta cycle, but typically changes by *180*° between entry and exit, and is correlated with both the relative distance travelled and time spent within a place field (i.e. the rate of phase precession is positively correlated with running speed) [10], [12], [71]. The firing rate of active place cells follows a Gaussian distribution, such that maximum firing rate occurs around the centre of the place field [10]. In CA3, the mean in-field firing rate of place cells is ∼*15*Hz, although this is strongly modulated by various non-spatial cues [14], [66], [68].

There is considerable debate regarding the mechanisms of phase precession in place cells, fuelled by apparently contradictory empirical findings [72]. Here, we are more directly concerned with the manner in which theta coded neural dynamics interact with local plasticity rules in order to mediate the learning and recall of auto- and hetero- associative connections between active neurons. Hence, the phase at which simulated neurons in our network model fire is primarily dictated by external excitatory input, although it is important to note that phase precession has been empirically observed in both the dentate gyrus and entorhinal cortex, which constitute the two principal synaptic inputs to CA3 [12], [73]. Furthermore, detailed biophysical simulations suggest that input from these afferent structures plays a significant role in dictating the neural dynamics observed in CA3 [74], [75].

**Figure 1:**
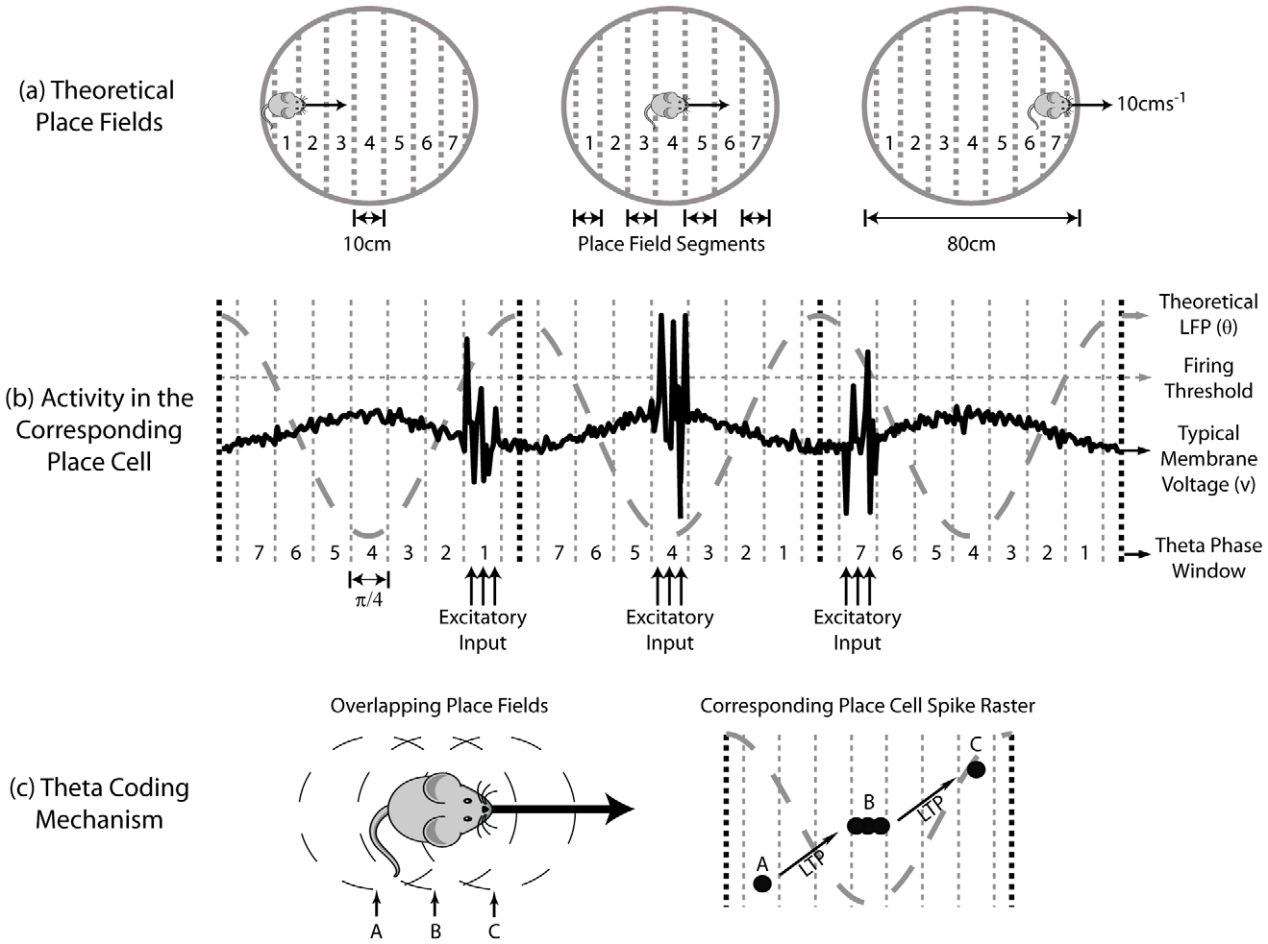
The Phenomenological Phase Precession Model and Theta Coding Mechanism. (**a, b**) Each theoretical place field and theta cycle (as defined by the value of *θ*) are divided into eight equally sized sub-sections. At each millisecond time step, the theoretical position within a place field dictates the theta phase window during which the corresponding place cell receives external excitatory input. Hence, when the theoretical animal enters a place field (segment 1), the corresponding place cell receives external stimulation late in the theta cycle (phase window 1); in the centre of the place field (segment 4), the corresponding place cell receives external, excitatory stimulation in the middle of the theta cycle (phase window 4); and as the place field is exited (segment 7), the corresponding place cell receives external, excitatory stimulation early in the theta cycle (phase window 7). The interplay of this external, excitatory stimulation with the constant, oscillatory inhibitory input to each place cell directs place cells to fire complex spike bursts when theoretical position is near the centre of the place field, and single spikes upon entry to or exit from the place field. Importantly, the random distribution of both inhibitory and excitatory inputs to each place cell produce stochastic firing activity within the corresponding phase window, such that place cells which encode for the same place field will fire with the same mean phase, but not necessarily in the same millisecond time step(s). (**c**) The phenomenological phase precession model creates a theta coding mechanism, whereby the sequence of place fields being traversed on a behavioural time scale is represented by a compressed sequence of activity in the corresponding place cells, repeated in every theta cycle.

During learning simulations, each place field is arbitrarily divided into eight equally sized sub-sections, and theta oscillations in the LFP (as defined by the value of *θ*) are similarly divided into subsections of *π/4* (between *π/8* and *15π/8*). At each millisecond time step, the theoretical position within a place field dictates the theta phase window at which the corresponding place cell receives external excitation, randomly sampled from a normal distribution with mean *I_ext_* and standard deviation *σ_ext_* (Figure 1a, b). This phenomenological model dictates that the mean phase of (stochastic) activity in place cells recedes in a step-wise fashion as the corresponding place field is traversed. In the majority of simulations, values of *I_ext_ = 5* and *σ_ext_ = 22.5* are used to generate a mean in-field firing rate of ∼*15*Hz, with active place cells tending to fire bursts at the peak of the LFP (as defined by the value of *θ*) and single spikes on the ascending and descending slope [10]. In other simulations, values of *σ_ext_* = [*12.5 ; 32.5 ; 42.5 ; 52.5*] are used to generate a range of mean in-field firing rates.

In these simulations, hypothetical place fields are generally *80*cm in diameter and traversed at a rate of *10*cms^−1^. Although this place field size is larger than that typically observed *in vivo* [10], [12], these values are chosen for computational convenience such that active place cells fire stochastically in each theta phase window for a period of *1*s before receding. Simulations were also performed using place field diameters of [*10cm ; 20cm ; 40cm*] – which effectively reduces the duration of time for which each theta coded stage of the learned sequence is applied to the network – and the only significant effect observed was a decrease in the rate of synaptic weight change (data not shown).

### The Synaptic Plasticity Model

The phase precession of place cells in the hippocampus produces a compressed, theta coded, sequence of firing within each oscillatory cycle that corresponds to the sequence of overlapping place fields being traversed on a behavioural timescale Figure 1c; [12], [13], [34]. These firing patterns are ideally suited to induce the long-term potentiation (LTP) and depression (LTD) of synapses by spike-timing dependent plasticity (STDP), and there is evidence that synaptic connections between overlapping place cells in rat hippocampus are potentiated during exploration [47]–[49], [76]. Mathematically, with *s = t_post_−t_pre_* being the time difference between pre- and post- synaptic spiking, the change in the weight of a synapse (Δ*w*) according to a standard STDP rule can be calculated using Equations 2.1–2.5 [77]–[82].
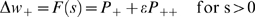
(2.1)

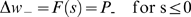
(2.2)

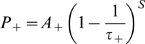
(2.3)

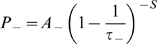
(2.4)

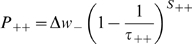
(2.5)The parameters *A_+_* and *A_−_* correspond to the maximum possible change in synaptic weight per isolated spike pair, while *τ_+_* and *τ_−_* denote the time constants that approximate an exponential decay of potentiation and depression increments respectively. The co-efficient *ε* determines the contribution of an additional potentiation process, which is equal to a trace of the most recent weight decrease at a synapse (with *s_++_ = t_Δw+_−t_Δw−_*) decaying exponentially with a time constant *τ_++_*. This term accounts for experimental observations of STDP in the hippocampus obtained using triplets of pre- and post- synaptic spikes, which suggest that depression is suppressed by potentiation within a short temporal window [48]. In accordance with empirical data, coincident pre- and post- synaptic firing elicits maximal depression from all STDP implementations examined here [49].

Previous auto-associative network models of hippocampal mnemonic function have most frequently utilised rate-coded Hebbian learning rules [16], [17], [22], [23], [83] – which typically dictate that changes in synaptic strength are proportional to the product of pre- and post- synaptic firing rates (*r_j,i_*) scaled by a learning rate *k* (Equation 3.1). This form of synaptic plasticity generates no competition between inputs or outputs of a single neuron, as any increase in synaptic weight produces an increase in post-synaptic firing rate in a positive feedback loop [84]. The BCM model (Equation 3.2) was proposed to address this issue, and postulates the existence of a theoretical modification threshold (*θ_m_*) that distinguishes between depression (at lower firing rates) and potentiation (at higher firing rates). The value of *θ_m_* is itself a function of pre- or post- synaptic activity, generating competition between synaptic inputs by making potentiation more difficult to achieve as the long-term average firing rates increases [85].
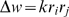
(3.1)

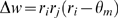
(3.2)Interestingly, it has been demonstrated that STDP can provide inherent competition using only local synaptic variables, and thus stabilise Hebbian learning processes [80], [82]. However, these properties rely on synaptic weights being either depressed or unchanged following an increase in pre-synaptic stimulation, which directly contradicts empirical data and the requirements of rate-coded associative learning. Conversely, several computational studies have described conditions under which STDP can be reconciled with the BCM formulation [77]–[79], [81]. This requires that the plasticity rule exhibit an increasing dominance of potentiation processes as inter-spike intervals (ISIs) are reduced [77]. Pair-based STDP rules, which assume a linear integration of potentiation and depression processes, require constraints to be placed on the nature of spike pair interactions and parameters that define the asymmetric learning window [77], [78], [81]. Triplet-based STDP rules, which explicitly account for the observed non-linear integration of potentiation and depression processes, dictate that mean synaptic weight increases with mean stochastic firing rate irrespective of the finer details of the STDP rule [77], [79].

We examine three different additive STDP implementations here, in order to draw a comparison between the emergent synaptic dynamics produced by each. The ‘BCM type’ pair- and triplet- based STDP rules have parameter values described in previous studies as allowing a reconciliation with rate-coded Hebbian learning (*A_+_*>*A_−_* and *τ_+_*<*τ_−_*), which also concur with empirical measurements made in the hippocampus [47], [49], [78], [81]. Conversely, the ‘non-BCM type’ pair-based STDP rule has parameter values noted in previous modelling studies for the generation of synaptic competition (*A_+_*<*A_−_* and *τ_+_* = *τ_−_*) [80], [82]. For each of these STDP rules, a lax nearest neighbour spike pairing scheme – which dictates that values of *P_±_* are reset to the value of *A_±_* upon afferent or efferent firing – is employed. Values of *A_±_* are also scaled by the value of *w_max_* such that ∼*60* spike pairings are sufficient to traverse the range of possible synaptic weight values, in accordance with empirical data [47]–[49]. The full details of each plasticity model examined are given in Table 1.

**Table 1:** Computational details of the STDP rules.

	STDP Rule	A_+_	A_−_	τ_+_ (ms)	τ_−_ (ms)	τ_++_ (ms)	ε
(A)	Pair Based BCM type	0.02*w_max_*	−0.01*w_max_*	20	50	N/A	0
(B)	Triplet Based BCM type	0.02*w_max_*	−0.01*w_max_*	20	50	20	1
(C)	Pair Based Non-BCM type	0.02*w_max_*	−0.021*w_max_*	20	20	N/A	0

Empirical evidence indicates that the degree of synaptic plasticity incurred by consistent stimulation protocols differs across the theta cycle, with potentiation incurred by burst pairings at the peak and depression (or de-potentiation) incurred by burst pairings at the trough of the LFP [86]–[89]. Here, we examine the effects of three different forms of theta modulated plasticity, for comparison, the details of which are shown in Equation 4.
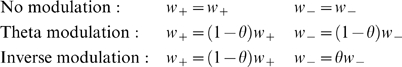
(4)In all simulations, hard limits are placed on the achievable strength of synapses, such that synaptic weights are maintained continuously in the range [*0 : w_max_*]. While there is little clear biological basis to inform the relative scale of synaptic weights, it is known that recurrent synapses in the CA3 region are generally incapable of solely provoking post-synaptic activity [90]. In order to generate an action potential using the neural dynamics employed here, a single synaptic current of *I = 16.5* is required, and therefore the value generally assigned to the maximum weight limit in these simulations is *w_max_ = 1*. In each simulation, all synaptic connections in the network are initialised with a weight of *0.01w_max_*.

### Neuromodulation and Recall

The neuromodulatory effects of Acetylcholine (ACh) have been hypothesised to separate periods of learning and recall in the hippocampus in order to avoid issues of interference [35], [91], [92]. Cholinergic input from the septum, terminating on local interneurons, can induce theta frequency oscillations in the CA3 region, facilitate LTP and enhance afferent input from the dentate gyrus and entorhinal cortex while suppressing recurrent excitation from intrinsic connections – thereby creating the ideal conditions for learning external associations via theta coding [92]. In the absence of cholinergic input pyramidal neurons in CA3 are disinhibited, synaptic plasticity is suppressed, and neural dynamics enter a state of large-amplitude irregular activity (LIA). During this period, postulated recall activity is observed in the form of sharp wave ripples (SWR) – short periods of high frequency firing in large populations of neurons with fine temporal structure that last ∼*100*ms and originate in CA3 [50]–[53], [69].

In our model, an abstract, global ACh signal modulates the scale of recurrent excitation and synaptic plasticity in the network throughout all simulations. The hypothetical concentration of ACh maintains a dimensionless value of *Φ = 1* during periods of theta coded learning and falls to a lower value during periods of recall. In both cases, the relative magnitude of recurrent synaptic weights in the network is scaled by a factor of *1/Φ*, while the magnitude of synaptic weight change is scaled by a factor of *Φ*. During periods of recall, theta frequency inhibitory input to the network is ceased and superthreshold excitation of magnitude *I_cue_ = 30* is provided to a small number of randomly selected neurons for a single millisecond time step. Subsequent activity - dictated by recurrent excitation alone - can then be compared to the auto- and hetero- associative correlations present in external input during learning and SWR activity observed *in vivo*. In these simulations, the effective speed of recall is strongly dependent on the size and overlap of place fields, as a reduction in place field size and offset implies a reduction in the total length of the learned route, such that the same temporal compression of recall firing equates to a slower traversal of that route.

We use several different measures to assess the fidelity of putative recall activity in this model. For hetero-associative and dual coded activity patterns, we examine the timing of the first action potential fired by each simulated place cell: firing before the first action potential in any place cell encoding for the following place field on the learned route is considered to be accurate, firing at the same time as the first action potential in any place cell encoding for the following place field on the learned route is treated indifferently, and failure to fire or firing after the first action potential in any place cell encoding for the following place field on the learned route is considered to be erroneous. For auto-associative patterns, we examine firing in all simulated neurons for a period of *20*ms following the external stimulation of a subset of ‘cued’ neurons from one of the learned activity patterns. Firing in any of the neurons from that learned pattern which are not externally stimulated (‘uncued’) during this period is considered to be accurate recall, while activity in any neuron that is not part of that pattern is considered to be erroneous. The Mann-Whitney U test is used to assess the significance of differences in the strengths of disparate populations of synaptic connections throughout this paper.

## Results

The aim of this study is to examine the encoding and subsequent reactivation of rate, temporal and dual coded activity patterns in a spiking recurrent neural network inspired by the neurobiology of CA3. Firstly, we compare the synaptic dynamics produced by three different STDP rules during theta coded learning: two ‘BCM type’, which can replicate the properties of rate-coded Hebbian learning (see Equation 3); and one ‘non-BCM type’, which has been demonstrated to generate competition between synapses and thereby stabilise Hebbian learning processes [77]–[82]. We also examine the effects of three dynamic plasticity modulation schemes, motivated by the empirical observation that identical stimulation protocols can induce significantly different changes in synaptic strength depending on their timing relative to the ongoing theta oscillation [86]–[89]. We demonstrate that, under certain conditions, STDP can encode both symmetric and asymmetric connectivity patterns between neurons that fire at concurrent or consecutive theta phase respectively. Secondly, we describe the putative recall dynamics generated in this network model, whereby superthreshold stimulation of a small number of randomly selected neurons following learning produces both pattern completion and sequence prediction via recurrent excitation.

### Theta Coded Hetero-Associative Learning

Lesions of the hippocampus have been demonstrated to disrupt the temporal ordering of information in memory, impairing recall of a sequence of locations visited [93], [94], olfactory cues presented [4], [95], [96], and trace eyeblink conditioning performance [97], [98]. This has led to the theory that the hippocampus – which exhibits sparse connectivity, temporally asymmetric synaptic plasticity, and theta coded neural dynamics - is critical for sequence learning and predictive recall [13], [28]–[37]. Hence, in the first set of simulations, we examine the learning of theta coded activity patterns in single neurons.

**Figure 2:**
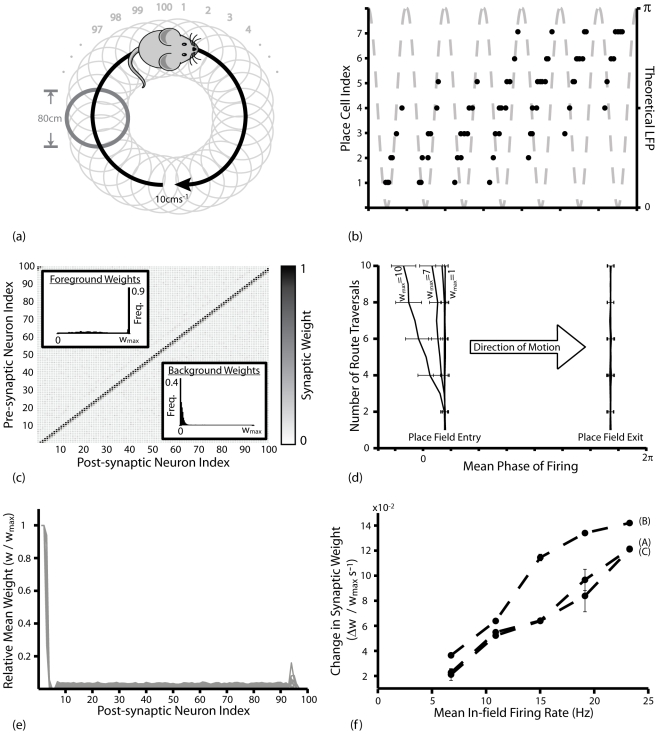
Theta Coded Hetero-associative Learning in a Spiking Recurrent Neural Network. (**a**) Theoretical details of theta coded hetero-associative learning simulations. *100* equidistant and overlapping place fields of *80*cm diameter, offset by *10*cm, form a circular route that is traversed repeatedly at a constant speed of *10*cms^−1^. Each place field is encoded by the activity of a single place cell. (**b**) Typical spike raster in seven representative place cells with consecutive and overlapping place fields, showing theta coded neural dynamics generated by the phenomenological phase precession model. For illustrative purposes, this figure was generated with much smaller place fields (*10*cm diameter) such that active place cells fire in each theta phase window for one oscillatory cycle only. (**c**) Typical synaptic weight matrix following learning. Asymmetric connections between place cells which correspond to consecutive place fields on the learned route are selectively and significantly potentiated. Inset: synaptic weight histograms for foreground and background connections (i.e. between a neuron and those that encode for either the three successive place fields on the learned route, or all other neurons in the network respectively). Data illustrated for the triplet based BCM type STDP rule with no plasticity modulation. (**d**) Mean phase of firing in all place cells at place field entry and exit on successive traversals of the route, averaged over *50* separate simulations. This demonstrates the asymmetric expansion of place fields against the direction of motion during spatial learning. Data illustrated for the triplet based BCM type STDP rule with no plasticity modulation. (**e**) The relative mean weight of synaptic connections between place cells in typical simulations with every combination of STDP rule and plasticity modulation scheme examined. The value of the post-synaptic neuron index corresponds to the distance – in place fields – between the pre- and post- synaptic place cell. (**f**) The mean rate of synaptic weight change at synapses connecting each place cell to that immediately ahead of it on the theoretical route averaged over *50* separate simulations, which correlates with mean in-field firing rate for (A) the pair-based BCM type; (B) the triplet-based BCM type; and (C) the non-BCM type STDP rule. Data illustrated for simulations with theta modulated plasticity, and synaptic weight change averaged over all neurons until synaptic weights saturate at the upper bounds.

In these simulations, place cell firing corresponds hypothetically to ten traversals of a route of one hundred equidistant and overlapping place fields of *80*cm diameter, each encoded by a single neuron, at a constant speed of *10*cms^−1^ (Figure 2a). However, this form of activity could just as easily correspond to a temporal sequence of non-spatial stimuli encountered on a behavioural timescale [29], [40], [64]. The spike raster shown in Figure 2b is representative of the neural dynamics generated by the phenomenological theta coding model, which replicates the gross features of phase precession observed in the hippocampus *in vivo*.

Figure 2c illustrates the typical asymmetric weight matrix that develops – with connections between each place cell and those that follow it on the theoretical route being selectively and significantly potentiated to create a bi-modal distribution of synaptic strengths (inset). Figure 2d illustrates the asymmetric expansion of place fields that proceeds over the course of these simulations, a phenomenon that has been observed experimentally [99]. This results from an increase in excitatory input to each place cell from those preceding it on the route as recurrent connections are potentiated, and the magnitude of place field expansion is therefore correlated with the value assigned to the maximum excitatory synaptic weight (*w_max_*).

It is important to note that the particular details of the STDP rule utilised here makes little difference to the efficient learning of hetero-associative sequences (Figure 2e). Furthermore, the strength of asymmetric connections saturates at the upper bounds regardless of whether neurons fire bursts or single spikes throughout each theta cycle – although mean in-field firing rate is correlated with the rate of synaptic weight change (Figure 2f). These results demonstrate that the combination of theta coding and STDP in a spiking recurrent network is sufficient to mediate rapid and robust sequence learning, irrespective of the finer details of the plasticity rule, in accordance with several previous models [12], [13], [28]–[37].

### Theta Coded Auto-Associative Learning

Although the majority of electrophysiology studies have focused on spatial memory, there is a growing body of evidence to suggest that non-spatial stimuli are also encoded in the activity of single neurons in the hippocampus and can significantly modulate the firing rate of established place cells [4]–[7], [9], [14], [57], [68], [100]. Computational theories of episodic memory function generally posit that discrete patterns of rate-coded activity, corresponding to the conjunctively coded sensory elements that constitute an experience, are auto-associated in the recurrent connections of CA3. This cortical activity can subsequently be fully recreated from partial sensory cues via a process of pattern completion [16]–[23], [68], [101].

However, auto-associative network models of the CA3 region have often been criticised on the grounds of biological realism for failing to include realistic neural and synaptic dynamics [39], [102]. Furthermore, it has been suggested that the inherently asymmetric nature of STDP directly contradicts rate-coded associative learning, which explicitly depend on the development of strong bi-directional connections [13], [82], [ but see 41], [42], [44], [45], [77]. Here, we examine whether auto-associative learning can be achieved in a network model that incorporates the main features of neural and synaptic dynamics observed in CA3 – namely, phase precession and STDP.

**Figure 3:**
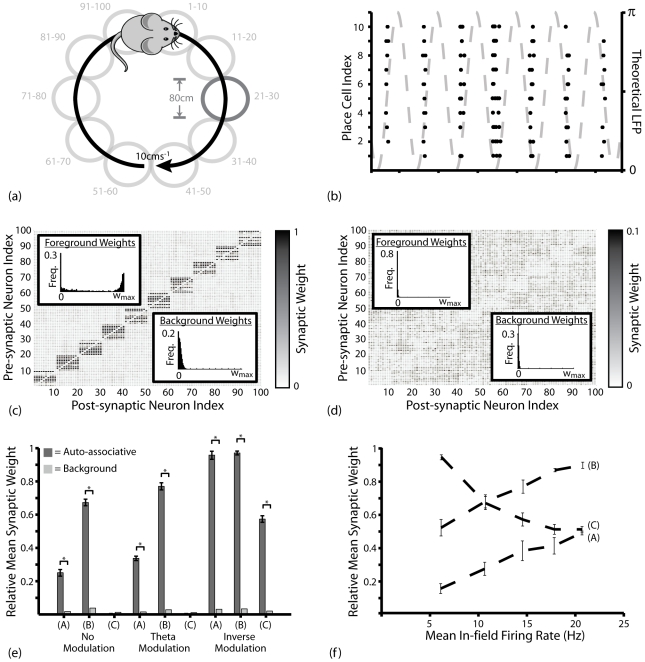
Theta Coded Auto-associative Learning in a Spiking Recurrent Neural Network. (**a**) Theoretical details of theta coded auto-associative learning simulations. *10* equidistant but non-overlapping place fields of *80*cm diameter, offset by *80*cm, form a circular route that is traversed repeatedly at a constant speed of *10*cms^−1^. Each place field is encoded for by ten place cells. This form of input effectively corresponds to repeated presentations of ten binary and orthogonal activity patterns. (**b**) Typical spike raster in place cells encoding for a single place field. For illustrative purposes, this figure was generated with much smaller place fields (*10*cm diameter) such that typical activity at each phase of theta can be seen more clearly. (**c**) Typical synaptic weight matrix following learning with the BCM type STDP rules, illustrating how connections between neurons that encode for the same place field are selectively and significantly potentiated. Data shown for triplet-based STDP with theta modulated plasticity. (**d**) Synaptic weight matrix following learning with the non-BCM type STDP rule and theta modulated plasticity. Under these conditions, synapses between place cells that encode for the same place field are depressed below the mean weight of other connections in the network. (**e**) The mean weight of synapses connecting each place cell to those that encode for the same place field (dark grey) and different place fields (light grey) following ten traversals of the theoretical route, averaged over *50* separate simulations, for the pair- and triplet- based BCM type STDP rules (A and B respectively) and the non-BCM type STDP rule (C). (**f**) The relative mean asymptotic weight of auto-associative connections averaged over *50* separate simulations, illustrating that the relative strength of auto-associative connections is positively correlated with mean in-field firing rate for (A) the pair based BCM type STDP rule (with theta modulated plasticity); and (B) the triplet based BCM type STDP rule (with theta modulated plasticity); but negatively correlated with mean in-field firing rate for (C) the pair based non-BCM type STDP rule (with inversely modulated plasticity).

In these simulations, input to the network effectively corresponds to ten presentations of ten binary and orthogonal activity patterns, in accordance with previous auto-associative network models [22], [23]. However, this form of input could also correspond to ten traversals of a route of ten non-overlapping place fields of *80*cm diameter, each encoded by the activity of multiple place cells, at a constant speed of *10*cms^−1^ (Figure 3a). The phenomenological phase precession model implemented dictates that neurons which are active in the same pattern (i.e. place cells that encode for the same place field) fire stochastically with equal mean phase, the value of which decreases in a step-wise fashion over the course of a single presentation (Figure 3b).

Our results demonstrate that successful auto-associative learning depends on a plasticity rule that produces net potentiation at the high instantaneous firing rates (i.e. short ISIs) present during near-synchronous firing in bi-directionally connected neurons [77]. Accordingly, both the pair- and triplet- based BCM type STDP rules selectively and significantly potentiate synaptic connections between place cells that encode for the same place field (Figure 3c, Mann-Whitney U-test, p<0.01). Conversely the non-BCM type STDP rule produces net depression of synaptic connections between concurrently active neurons (Figure 3d). This demonstrates that efficient auto-associative learning can be achieved in a spiking recurrent neural network when an STDP rule that can be reconciled with rate-coded Hebbian learning is employed, and that this function is fully compatible with theta coded neural dynamics created by the phase precession of principal cells *in vivo*.

However, in contrast to the hetero-associative learning simulations described above, the mean weight of auto-associative connections in simulations with BCM type STDP rules generally reaches an asymptote well below the upper bounds - exhibiting a bi-modal distribution (Figure 3c, inset) except where potentiation and depression processes are inversely modulated (Figure 3e). This is a consequence of the persistently alternating temporal order of spike pairings at these synapses, which produces an equilibrium between potentiation and depression processes. The position of this equilibrium is significantly affected by several features of the neural dynamics and synaptic plasticity rule employed. For example, the asymptotic mean weight of auto-associative connections increases with mean in-field firing rate for the BCM type STDP rules, but decreases with mean in-field firing rate for the non-BCM type STDP rule (Figure 3f). In both cases, the rate of synaptic weight change (whether positive or negative) correlates with the mean in-field firing rate.

**Figure 4:**
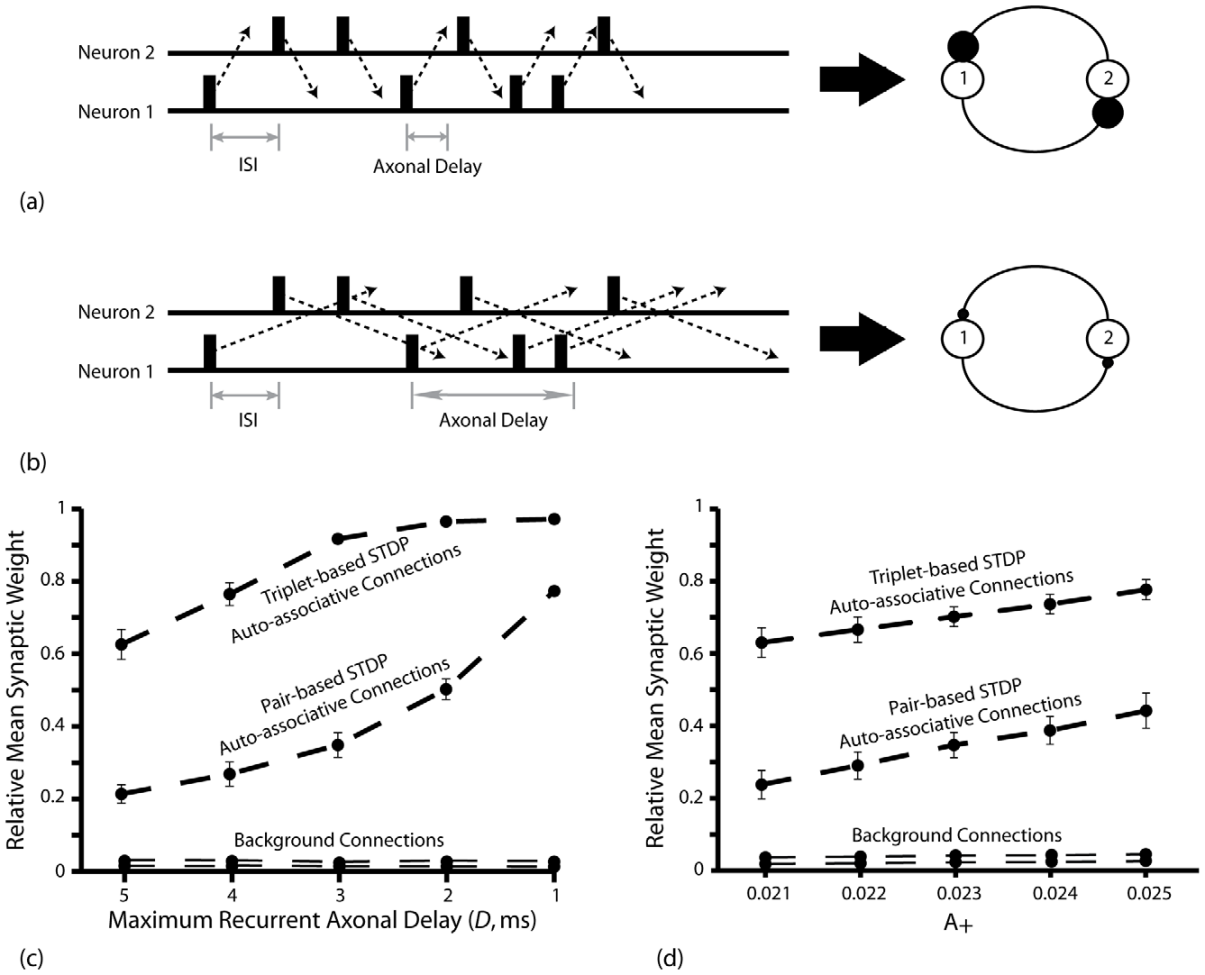
Effects of Axonal Delay and Profile of the Asymmetric Learning Window on Auto-associative Learning. (**a**) Action potentials in bi-directionally connected neurons are more likely to reach the pre-synaptic terminal before the end of synchronous (but stochastic) complex bursts, and therefore induce the potentiation of inter-connecting synapses, if axonal delays are shorter. (**b**) Conversely, action potentials in each simulated neuron are more likely to arrive at the pre-synaptic terminal after the end of synchronous (but stochastic) complex bursts, and therefore induce depression of the inter-connecting synapses, if axonal delays are longer. (**c**) Relative mean synaptic weight (*w*/*w_max_*) of auto-associative and background connections (i.e. between neurons that are in the same or different patterns respectively) produced by the BCM type STDP rules following ten traversals of the theoretical route described in Figure 3a with a varying scale of axonal delays (*D*). Data is averaged over *50* separate simulations. (**d**) Relative mean synaptic weight (*w*/*w_max_*) of auto-associative and background connections produced by the pair- and triplet- based BCM type STDP rules following ten traversals of the theoretical route described in Figure 3a with varying values of A_+_ and therefore different positions of the theoretical modification threshold (*θ_m_*). Data is averaged over *50* separate simulations.

In these simulations, spike pairing events that dictate changes in synaptic strength do not take place immediately following firing, but rather once an action potential reaches the pre-synaptic terminal. Hence, the range of axonal delays (*D*) can also have a significant impact on the relative strength of auto-associative connections. In bi-directionally connected neurons exhibiting near-synchronous bursting, longer axonal delays imply that the arrival of spikes at pre-synaptic terminals is more likely to occur after post-synaptic firing and therefore generate depression; while shorter axonal delays imply that afferent spikes are more likely to precede post-synaptic activity and therefore generate potentiation (Figures 4a, b). Accordingly, for the BCM type STDP rules examined here, both the rate of potentiation and asymptotic mean weight of auto-associative connections increase as the scale of axonal delays is decreased (Figure 4c). This also explains why, in Figure 3c, the strength of auto-associative post-synaptic connections formed by some place cells is uniformly weak, as the axonal delay of that neuron is higher than others encoding for the same place field.

Similarly, for BCM type STDP rules, parameters that dictate the profile of the asymmetric learning window (*A_±_* and *τ_±_*) effectively define the position of the theoretical modification threshold (*θ_m_*, Equation 3.2) that marks the transition between net synaptic depression (at low stochastic firing rates) and potentiation (at high stochastic firing rates) [41]–[43], [64]. Hence, lowering the theoretical modification threshold – by increasing the value assigned to *A_+_*, for example – produces a greater degree of potentiation at set in-field firing rate, and therefore increases the asymptotic mean weight of auto-associative connections in these simulations (Figure 4d).

### Dual Coded Learning

It has been proposed that the sequential co-activation of groups of neurons during behaviour can be encoded via Hebbian plasticity [55], [56]. Subsequently, transient activity patterns in the same cell assembly can be initiated by internal cognitive processes and maintained via mutual excitation. Phase precession in ensembles of place cells encoding for overlapping place fields represents a prominent empirical model of cell assembly dynamics in the brain [50], [57], [59]. However, previous models of hippocampal mnemonic function have generally focussed on the learning and recall of either discrete rate-coded or sequential temporally-coded activity patterns, while few studies have attempted to integrate these computational models within a single framework [27], [39], [42]–[44]. Here, we demonstrate that both auto- and hetero- associative learning can proceed simultaneously in our network model, such that repeatedly synchronous firing with weak sequence bias produces bi-directional connections while repeatedly asynchronous firing produces asymmetric connections.

**Figure 5:**
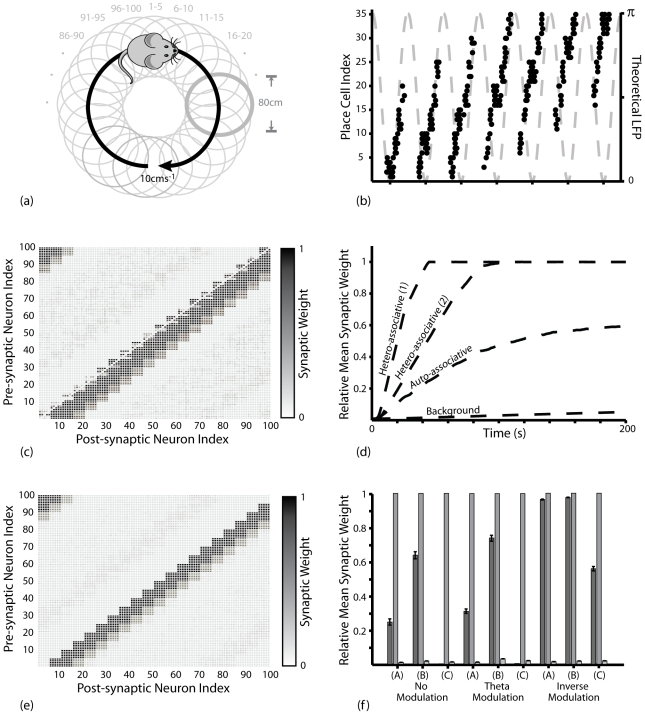
Dual Coded Learning in a Spiking Recurrent Neural Network. (**a**) Theoretical details of dual coding simulations. *20* equidistant and overlapping place fields of *80*cm diameter, offset by *10*cm, form a circular route that is traversed repeatedly at a constant speed of *10*cms^−1^. Each place field is encoded by five place cells. (**b**) Representative spike raster in thirty-five place cells encoding for seven separate but overlapping place fields. Place cells encoding for different place fields fire stochastically within different theta phase windows. (**c**) Typical synaptic weight matrix following ten traversals of the route for the BCM type STDP rules. Synaptic connections between place cells that encode for successive place fields on the theoretical route saturate at the upper weight bounds and synaptic connections between place cells that encode for the same place field are selectively and significantly potentiated. Data illustrated for triplet-based STDP with theta modulated plasticity. (**d**) Dynamic changes in the relative mean weight (*w*/*w_max_*) of auto-associative (between place cells encoding for the same place field), hetero-associative (between place cells encoding for a place field and that either one or two steps further along the route), and background (between place cells and those encoding for place fields not within three steps further along the route) connections. Data illustrated is for the triplet-based BCM type STDP rule with theta modulated plasticity. (**e**) Typical synaptic weight matrix following ten traversals of the route when the non-BCM type STDP rule is employed with theta modulated plasticity. In contrast to (c), auto-associative connections between place cells that encode for the same place field are depressed, while hetero-associative connections between place cells that encode for successive place fields saturate at the upper weight bounds. (**f**) The relative mean weight of synapses connecting each place cell to those that encode for the same place field (dark grey), the next place field on the learned route (medium grey), and all place fields not within three steps ahead on the learned route (light grey) following ten traversals, averaged over *50* separate simulations, with the pair- and triplet- based BCM type STDP rules (A and B respectively) and the non-BCM type STDP rule (C).

Input to the network during these simulations corresponds to a route of twenty overlapping place fields of *80*cm in diameter, each encoded for by five place cells, being traversed at a constant speed of *10*cms^−1^ (Figure 5a). This form of input is equivalent to the repeated presentation of a sequence of binary orthogonal activity patterns [39]. Figure 5b illustrates a representative spike raster observed during these simulations, demonstrating how the phenomenological phase precession mechanism dictates that place cells encoding for the same place field fire stochastically within the same theta phase window while place cells encoding for successive place fields fire in successive theta phase windows.

Figure 5c illustrates the typical synaptic weight matrix that develops during simulations with BCM type STDP rules, where synapses connecting each hypothetical place cell to those that encode for the same or successive place fields are rapidly, selectively and significantly potentiated (Figure 5d). Conversely, when the non-BCM type STDP rule is utilised (and potentiation and depression are not inversely modulated), then hetero-associative sequence learning proceeds robustly but strong, bi-directional auto-associative connections are not generated (Figure 5e). In fact, there is no significant difference in the asymptotic mean weight of auto- or hetero- associative connections generated in any of these dual coding simulations and those described above with equal parameter values (Figures 2e, 3e, f, 4c, d; 5f). The experimentally observed asymmetric expansion of place fields against the direction of motion during spatial learning (Figure 2d) also proceeds during dual coded learning simulations (data not shown).

These results again demonstrate that an STDP rule which exhibits a dominance of potentiation at short ISIs – and can therefore mediate rate-coded Hebbian learning – is essential for efficient auto-associative learning to proceed. However, each of the BCM type STDP rules examined here exhibits a significant functional weakness: background connections (i.e. synapses between neurons which are not in the same or immediately successive patterns) undergo slight but continual potentiation throughout all simulations, indicating a lack of inherent synaptic competition (Figure 5d). Effectively, a positive feedback loop arises between the potentiation of a synapse and a reduction in the latency of post-synaptic firing following an identical pre-synaptic input. This lack of competition may be necessary to allow the development of strong, bi-directional connections using the asymmetric STDP rule, as the mean weight of background connections correlates with that of auto-associative connections in all simulations (Figure 5f), but is also reminiscent of the global stability issues commonly encountered by rate-coded Hebbian learning [84].

### Recall Phase

Electrophysiology studies have demonstrated that learned routes – corresponding to the theta coded activity patterns observed in place cells during exploration – are pre-played in sharp wave ripples (SWR) at the beginning of (and during) a journey, replayed in reverse order at the end of a journey, and replayed in the original order during sleep [51]–[53]. The temporal order and relative latency of firing observed during exploration is preserved during this rehearsal and replay activity, which suggests a Hebbian learning mechanism on the timescale of STDP [50], [54]. Here, we examine the recall activity generated by recurrent excitation in our network under similar conditions - when theta frequency inhibitory input is ceased, the hypothetical concentration of ACh is reduced (to modulate the magnitude of recurrent excitation and synaptic plasticity), and superthreshold external excitation is applied to small numbers of neurons. This activity can then be compared to both the auto- and hetero- associations created during learning in the simulations described above and SWR activity observed *in vivo*.

**Figure 6:**
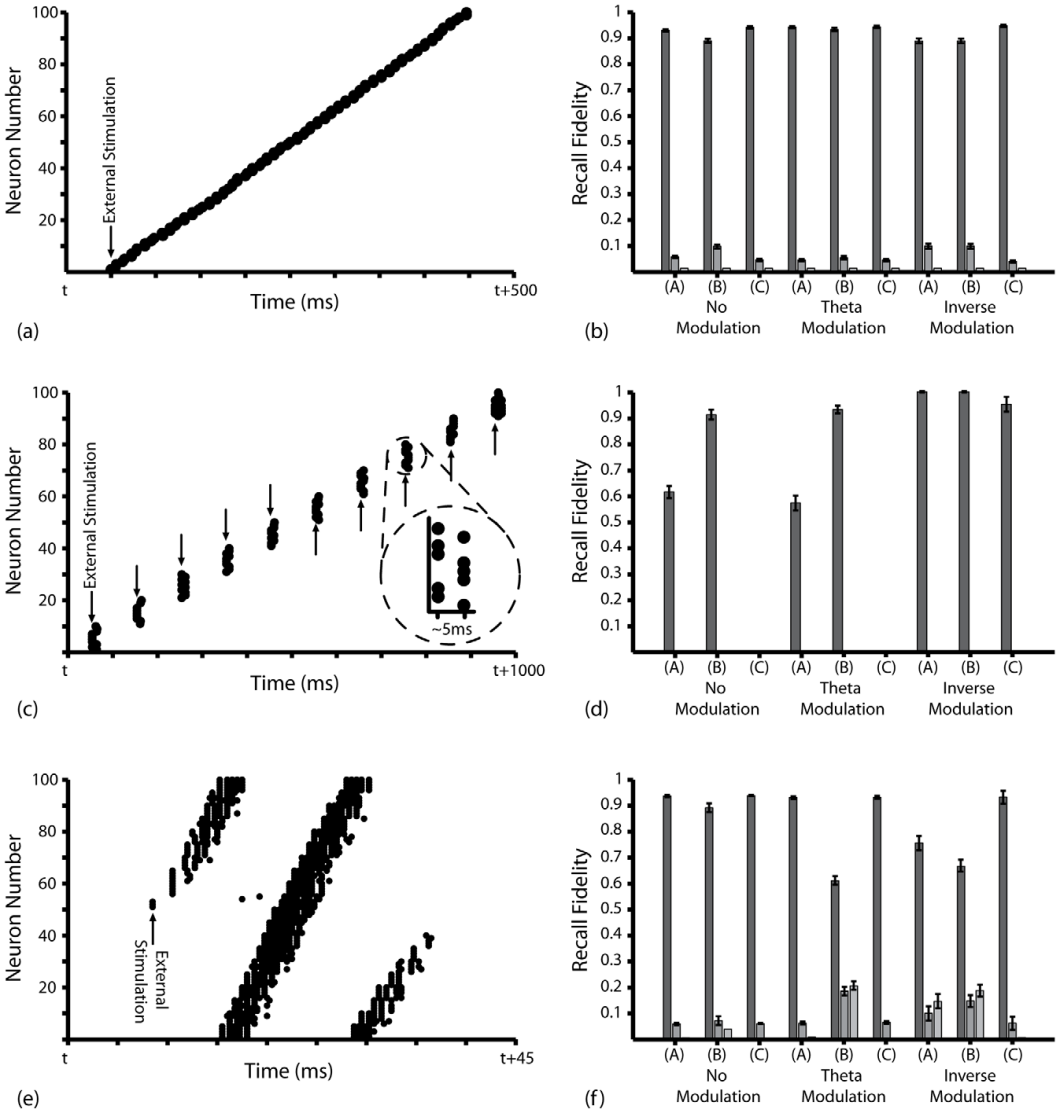
Putative Sharp Wave Ripple Recall Activity Following Theta Coded Learning. (**a**) Typical spike raster observed in the network during recall simulations following hetero-associative learning (as described in Figure 2). Externally stimulated firing of a single neuron produces sequential recall activity in all neurons that constitute the originally learned pattern; (**b**) Statistics relating to hetero-associative recall for each STDP rule and plasticity modulation scheme examined. Figures shown represent data averaged over *1000* randomly initialised recall epochs with *Φ* = *0.05* following hetero-associative learning simulations with the (A) pair-based BCM type; (B) triplet-based BCM type; (C) pair-based non-BCM type STDP rules. Data illustrated for the relative frequency of neurons that fired before (dark grey); at the same time as (medium grey); and after (light grey) the simulated neuron encoding for the next place field on the learned route. (**c**) Typical spike raster observed in the network during recall simulations following auto-associative learning (as described in Figure 3). External stimulation of a random subset of (cued) neurons from each learned pattern (five out of ten, in this case) generates selective firing in (uncued) neurons that encode for the same place field/pattern after *5–10*ms (depending on the plasticity rule employed during learning, and the concentration of ACh employed during recall). (**d**) Statistics relating to auto-associative recall for each STDP rule and plasticity modulation scheme examined. Figures shown represent data averaged over *1000* randomly initialised recall epochs following learning with the (A) pair-based BCM type STDP rule, and *Φ* = *0.05*; (B) triplet-based BCM type STDP rule, and *Φ* = *0.083*; (C) pair-based non-BCM type STDP rule, and *Φ* = *0.05*. Data illustrated for the relative frequency of uncued neurons that fire within *20*ms of externally cued activity in other neurons within the same pattern (dark grey) and the relative frequency of neurons in different, uncued patterns that fire within the same temporal window (light grey). (**e**) Typical spike raster observed in the network during recall simulations following dual coded learning (as described in Figure 5). External stimulation of a random subset of neurons from a single pattern (three out of five, in this case) produces sequential recall activity in simulated neurons that encode for each successive place field on the learned route. This neural activity pattern is reminiscent of sharp wave/ripple dynamics observed during putative recall activity in the hippocampus; (**f**) Statistics relating to dual coded recall for each STDP rule and plasticity modulation scheme examined. Figures shown represent data averaged over *1000* randomly initialised recall epochs with *Φ* = *0.111* following dual coded learning for the (A) pair-based BCM type; (B) triplet-based BCM type; and (C) pair-based non-BCM type STDP rules. Data illustrated for the relative frequency of neurons that fired before (dark grey); at the same time as (medium grey); and after (light grey) the first action potential in any simulated neuron encoding for the next place field on the learned route.

Firstly, we examine sequence prediction following hetero-associative learning. As illustrated by Figure 6a, superthreshold stimulation of a single, randomly selected neuron typically produces accurate sequential firing in all neurons that constitute the original learned pattern over a period of ∼*400*ms. Over one thousand separate recall epochs, the fidelity of recall activity produced is typically ∼*90*% for every STDP rule and plasticity modulation scheme examined (Figure 6b). The sequential firing patterns observed in these recall simulations continue indefinitely in the absence of inhibitory input to suppress the effects of recurrent excitation. This is a product of the fact that each neuron has few strong post-synaptic connections, and hence the concentration of ACh must be reduced to a level whereby the relative scale of recurrent synaptic weights allows single synapses to produce post-synaptic firing (*Φ = 0.05* in Figures 6a, b for example).

Secondly, we examine pattern completion following auto-associative learning by providing superthreshold excitation to random partial cues consisting of five out of ten simulated neurons from each learned pattern. As illustrated in Figure 6c, the uncued neurons in each pattern are typically activated by recurrent excitation shortly after externally cued activity while other neurons in the network remain silent. The fidelity of recall activity produced in these simulations reflects the relative strength of auto-associative connections generated during learning (Figures 3e; 6f). However, pattern completion does not rely on an ‘idealised’ weight matrix: >*90*% accurate recall activity is produced following learning with the triplet-based BCM type STDP rule, which produce a mean auto-associative weight of ∼*0.7w_max_*. Furthermore, no erroneous activity (i.e. firing in neurons that are not part of the cued pattern) is produced following learning with any of the STDP rules over a thousand separate recall simulations (Figure 6d).

Finally, we examine recall activity following dual coded learning by applying superthreshold stimulation to a randomly selected subset of simulated neurons (three out of five) that encode for a single theoretical place field on the learned route. As illustrated in Figure 6e, this generates sequential recall activity in all neurons encoding for each consecutive place field on the route over a period of ∼*33*ms. This activity is self-terminating and on approximately the same timescale as sharp wave ripples observed *in vivo*. Interestingly, strong auto-associative connections are not necessary to generate these sequential activity patterns in encoded place cell assemblies. Consistently high recall fidelity is produced following learning with the non-BCM type STDP rule, when only strong hetero-associative connections are generated (Figure 6f). In fact, the fidelity of recall activity is generally inversely correlated with the relative strength of auto-associative synaptic weights, regardless of the concentration of ACh employed.

**Figure 7:**
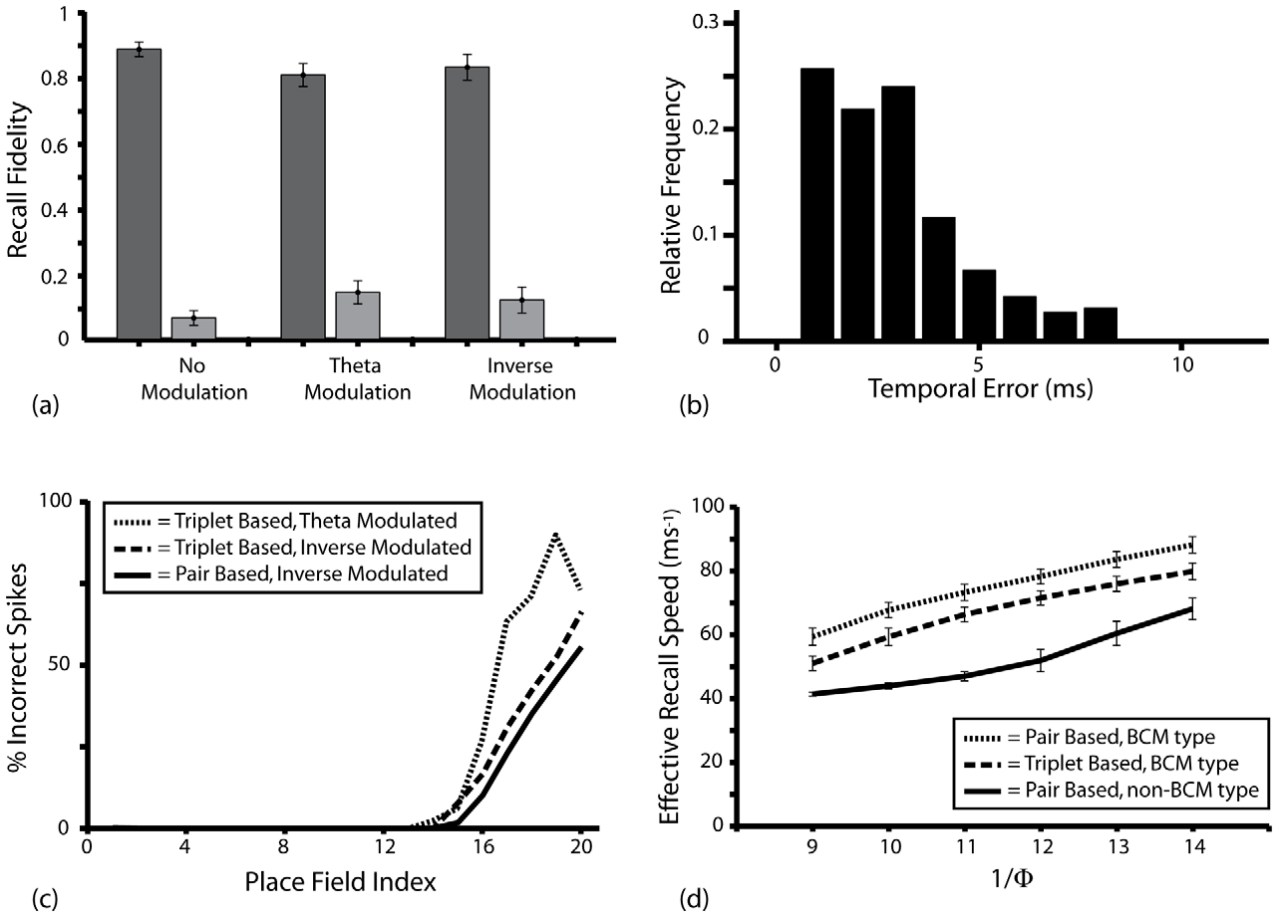
Further Details of Putative Sharp Wave Ripple Recall Activity. (**a**) Statistics relating to dual coded recall following learning with the triplet-based BCM type STDP rule, when all background connections (i.e. between place cells and those encoding for all place fields that are not within three steps on the learned route) are set to *0* following learning. Data shown for *Φ* = *0.111* and averaged over *1000* randomly initialised recall epochs, illustrating the relative frequency of neurons that fired before (dark grey); at the same time as (medium grey); and after (light grey) the first action potential in any simulated neuron encoding for the next place field on the learned route. This can be directly compared with Figure 6f. (**b**) Histogram of temporal magnitude for every erroneous spike fired during *1000* randomly initialised dual coded recall epochs with *Φ* = *0.111* following learning with the triplet-based BCM type STDP rule and theta modulated plasticity (that being the lowest recall fidelity displayed in Figure 6f). (**c**) The mean percentage of incorrectly timed recall spikes observed during sharp wave ripple recall activity, displayed in terms of the distance along the learned route, in place fields, from the externally stimulated place cells. Data is averaged over *1000* randomly initialised dual coded recall epochs for the BCM type STDP rules with *Φ* = *0.111*. (**d**) The effective speed of SWR activity – calculated using the time interval between the first spike caused by superthreshold external stimulation and the first subsequent spike in a place field encoding for the same place field following the propagation of activity along the entire length of the learned route – for different concentrations of ACh. Data is averaged over *1000* randomly initialised dual coded recall epochs, following learning with theta modulated plasticity.

However, further simulations demonstrate that the relative scale of background synaptic connections contributes more significantly to erroneous recall activity than that of auto-associative connections – as arbitrarily setting the weight of all background connections to zero following dual coded learning generally eliminates all incorrect firing activity during subsequent recall (Figure 7a). Furthermore, the temporal error in recall activity following dual coded learning with BCM type STDP rules is generally low (Figure 7b), such that correct sequence prediction might be produced if one considers only the mean time of firing in all neurons that encode for a single place field. It is also interesting to note that recall fidelity consistently decreases over time, with the vast majority of erroneous recall activity occurring in the final ∼*15*ms of each putative sharp wave ripple event (Figure 7c). Intuitively, the effective speed of putative SWR activity – calculated using the time taken for sequential activity to progress through place cells encoding for the entire length of the *2*m track – is significantly affected by the concentration of ACh present in the network (Figure 7d), which dictates the magnitude of recurrent synaptic currents. The effective speed of recall following hetero-associative learning simulations is significantly slower (∼*25*ms^−1^), due to the fact that fewer strong pre-synaptic connections (and therefore weaker recurrent synaptic currents) exist for each simulated place cell.

## Discussion

Recurrent neural networks have an established history in computational neuroscience as prototypical models of declarative memory function [16], [17], [22], [23]. It is widely accepted that the CA3 region of the hippocampus – which contains the densest recurrent connectivity in the brain, and wherein synaptic plasticity can be rapidly and reliably induced – represents their biological correlate [18]–[21]. Despite their success in replicating key features of spatial and declarative mnemonic function, these models have often been criticised for their lack of biological realism in failing to integrate neural and synaptic dynamics which correspond to those observed in the hippocampus [39], [102]. In contrast, we have presented a spiking recurrent neural network that utilises theta coded neural dynamics and STDP to encode and recall both rate and temporally coded input patterns. This integrates previous auto- and hetero- associative network models of the hippocampus within a single framework using a single plasticity rule and provides them with a firmer basis in modern neurobiology. The encoding and reactivation of dual coded cell assemblies – putative phase sequences of activity in mutually exciting ensembles of cells – is believed to represent a fundamental mechanism for cognitive processing [55], [56], [58].

Our findings demonstrate that, under certain biologically feasible constraints, the temporally asymmetric STDP rule can replicate rate-coded Hebbian learning by generating strong bi-directional connections between neurons firing at an elevated rate with no repeated sequence bias [77]–[79], [81]. This implies that STDP can support rate-coded auto-associative network function and mediate cognitive map formation during open field exploration [3], [16], [17], [20], [22], [23]. The critical condition upon which this dual rate- and temporally- coded learning relies is that the magnitude of potentiation exceeds the magnitude of depression incurred by spike pair interactions at shorter ISIs. For pair-based STDP rules, this requires temporal restrictions on spike pairing and constraints on the profile of the asymmetric learning window, which concur with empirical measurements in the hippocampus [47], [77], [78]. For triplet-based STDP rules, it is implicitly generated by the short-term dominance of potentiation which, interestingly, is on a similar timescale to the duration of a single theta cycle [48], [79]. Conversely, STDP rules which do not dictate a dominance of potentiation at short ISIs prevent the development of strong bi-directional connections, except where synaptic plasticity is modulated such that only potentiation can proceed at the peak of the LFP. Under these conditions, however, synaptic weights undergo net depression as mean in-field firing rate increases [80], [82].

Despite replicating the gross phenomenological features of rate- and temporally- coded synaptic plasticity data, the BCM type STDP rules examined here exhibit several emergent features that contradict empirical observations. Firstly, the additive nature of these plasticity rules generates bimodal weight distributions that are at odds with experimental measurements [103]. However, an additive STDP rule might better approximate the known bi-stability of synaptic strengths, and a unimodal distribution of maximum weight limits could account for their observed heterogeneity [104]. Previous computational modelling has also demonstrated that the synaptic dynamics produced by additive STDP rules can, under certain conditions, be qualitatively replicated by a multiplicative plasticity rule [77]. Secondly, empirical studies suggest that no depression is incurred at connections between place cells encoding for overlapping place fields *in vivo* [76]. In our model, a synaptic plasticity rule that accounts for this data would more fully potentiate auto-associative connections, although our results indicate that this is not necessary for efficient pattern completion. Furthermore, it is interesting to note that connections between place cells that encode for place fields with higher degrees of overlap appear to be more modestly potentiated *in vivo* see Figure 5G in [76].

Empirical studies of synaptic plasticity in the hippocampus have also demonstrated that the potentiation of asymmetric connections by STDP depends on post-synaptic bursting [46]. A plasticity rule that accounted for this data might therefore generate hetero-associative synaptic weights that rely explicitly on mean in-field firing rate, as observed for auto-associative connections in this study. This should allow the implications of rate re-mapping in pyramidal cells within CA3 – whereby the manipulation of non-spatial cues within an environment significantly modulates the firing rate of active place cells – to be examined [14], [68]. In this context, connections between place cells that exhibit high in-field firing rates during learning – indicating the current configuration of non-spatial stimuli within the corresponding environment – would be preferentially potentiated. During subsequent SWR activity, more complex transient dynamics within the global place cell assembly encoding for that environment might therefore be produced, according to the particular stimulus applied to the network and its relationship to previously encoded configurations.

From a functional standpoint, our findings suggest that the synaptic competition described in several previous theoretical studies as a putative homeostatic mechanism is absent for BCM type STDP rules [77], [80], [82]. This is not surprising, considering the wealth of literature regarding the global instability of rate-coded Hebbian learning mediated by purely local variables [84], [85]. However, it does imply that the encoding of multiple, overlapping cell assemblies – as opposed to the single episodes examined here - could rapidly lead to the saturation of synaptic weights and interference during recall. Some additional mechanism – such as synaptic scaling, weight normalisation or metaplasticity – is therefore required to guarantee the long-term efficiency of network models that use BCM type STDP rules by preventing the slow potentiation of all connections, particularly since the strength of background connections in our network model has been shown to correlate with erroneous recall activity [84], [85]. It is interesting to note that empirical data suggests a broad dissociation between net synaptic potentiation during waking and net depression during sleep [105]. Within the context of modelling mnemonic function, any mechanism of synaptic competition is likely to affect the emergent dynamics of learning and recall in terms of the long-term stability of previously encoded associations.

It is also useful to appraise the results presented here in terms of more general theories of hippocampal mnemonic function. The plasticity model implemented prevents the potentiation of synaptic connections between place cells corresponding to trajectories against the direction of motion, and therefore omits the possibility of reverse replay in encoded cell assemblies during sharp wave ripples [51], [54]. Interestingly, putative SWR activity in our simulations also proceeds an order of magnitude more quickly than that observed *in vivo* – with effective recall speeds of ∼*80*ms^−1^ (Figures 6e, 7d) compared to the ∼*8*ms^−1^ observed experimentally in CA1 [106]. Of course, the speed of SWR activity is strongly affected by estimates of place field size and overlap, which may differ significantly from the values used here. However, one critical abstraction in our network model may contribute to both the accelerated pace of SWR activity and the generation of erroneous recall activity following efficient dual coded learning, and that is the relative timescales of recurrent auto- and hetero- associative connections.

Previous theoretical research has suggested that processes of pattern completion and sequence prediction in CA3 must operate on different timescales in order to effectively differentiate between neural activity corresponding to different stages of a putative phase sequence, and it is not clear how this could be achieved in a single network with a fixed range of axonal delays. It has therefore been suggested that different regions of the hippocampus may mediate auto- and hetero- associative learning at distinct sets of synapses using a single plasticity rule, such as that presented here [39]. Our model suggests that CA3 can feasibly implement auto- and/or hetero- associative learning and recall. However, we have also demonstrated that auto-associative connections are not necessary for the reactivation of dual coded cell assemblies, and it seems plausible that purely hetero-associative dynamics could account for the putative function of CA3 in the rapid encoding of novel information and subsequent pattern completion [20], [21]. Conversely, it is possible that auto-associative connections exist within CA3, where relatively short axonal delays (which we have demonstrated to be necessary for auto-associative learning) are observed; while hetero-associative connections may be located in polysynaptic feedback connections between CA3 and the dentate gyrus. Activity corresponding to sharp wave ripples, which are believed to originate in CA3, have been documented in the dentate gyrus during sleep [107]. Identifying the loci of auto- and hetero- associative synaptic connections in the hippocampus remains an open problem for empirical neuroscience. It seems feasible that simultaneous recordings from these two regions and/or or the pharmacologically induced inhibition of firing in granule cells during sleep could elucidate the relative contribution of each region to the replay of previously learned associations.

The segregation of auto- and hetero- associative connections may also allow the reactivation of cell assemblies to proceed during encoding, rather than these processes being arbitrarily separated between different network states. Several converging strands of empirical research - as well as simple intuition - suggest that some element of prediction, based on prior experience, is present during periods of theta coded learning, including changes in place field geometry and predictive theta modulated activity in place cells at decision points on a maze task [65], [99], [108]–[110]. Indeed, it has been suggested that the phenomena of phase precession itself may be generated by self-propagating ‘recall’ activity in cell assemblies within the hippocampus [67]. In our model, hetero-associative connection delays are on the same timescale as those measured in inter-connected CA3 pyramidal cells (i.e. <5ms), and thus sequence prediction via recurrent excitation proceeds more quickly than theta coded activity corresponding to external input. It is possible that inhibition from different classes of interneuron, creating gamma oscillations within each theta cycle, and/or the modulated efficacy of recurrent excitation at different theta phases could selectively manipulate the timing of pyramidal cell firing [37], [39], [40], [43], [65]. Similarly, if the loci of hetero-associative connections are poly-synaptic feedback loops from the dentate gyrus, as discussed above, then the replay of sequences will be explicitly staggered and could therefore proceed between different (gamma) sub-cycles of the theta oscillation [39].

In summary, this research provides a synaptic plasticity rule that can mediate both rate and temporal coded learning within a spiking recurrent neural network. Furthermore, it provides an associative memory model that utilises this dual code in order to integrate the encoding and reactivation of both dynamic (spatial) and static (non-spatial) activity patterns. This allows manipulations of the plasticity rule, neuronal dynamics and neural network to be directly related to systems level function. Hebbian phase sequences of activity in mutually exciting cell ensembles, such as those examined here, have been postulated as a general mechanism of neural coding for cognitive processing [55], [58]. Support for this theory comes from recent empirical evidence from the hippocampus and pre-frontal cortex [57]–[60], [111], [112]. Furthermore, theoretical considerations are making it increasingly clear that cortical function cannot be characterised by fixed point attractor dynamics, and neural network models must therefore account for the transient dynamics observed *in vivo* [56]. This research provides a framework for an examination of how dual coded activity patterns could be encoded in recurrent synaptic connections and subsequently reactivated by ongoing internal or external dynamics.
